# Macroautophagy and normal aging of the nervous system: Lessons from animal models

**DOI:** 10.15698/cst2021.10.257

**Published:** 2021-10-06

**Authors:** Emmanouela Kallergi, Vassiliki Nikoletopoulou

**Affiliations:** 1 University of Lausanne, Department of Fundamental Neurosciences, Lausanne, Switzerland.

**Keywords:** proteostasis, macroautophagy, nervous system, aging

## Abstract

Aging represents a cumulative form of cellular stress, which is thought to challenge many aspects of proteostasis. The non-dividing, long-lived neurons are particularly vulnerable to stress, and, not surprisingly, even normal aging is highly associated with a decline in brain function in humans, as well as in other animals. Macroautophagy is a fundamental arm of the proteostasis network, safeguarding proper protein turnover during different cellular states and against diverse cellular stressors. An intricate interplay between macroautophagy and aging is beginning to unravel, with the emergence of new tools, including those for monitoring autophagy in cultured neurons and in the nervous system of different organisms *in vivo*. Here, we review recent findings on the impact of aging on neuronal integrity and on neuronal macroautophagy, as they emerge from studies in invertebrate and mammalian models.

## BRIEF OVERVIEW OF THE PROTEOSTASIS NETWORK: FOCUS ON NEURONAL MACROAUTOPHAGY

Neurons, as post-mitotic cells, cannot dilute out cellular debris, such as misfolded proteins or damaged organelles, simply by cell division. Therefore, maintaining cellular proteostasis is of paramount importance for their proper function and consequently for the integrity of the nervous system. Neuronal proteostasis is maintained by the coordinated action of protein synthesis, of chaperone-assisted protein folding and of two cellular degradation processes, namely lysosomal degradation and proteolysis by the ubiquitin-proteasome system (UPS). The roles of the UPS in neurons [1] and the effects of aging on UPS activity [2] have been extensively reviewed in the past and will not be covered here.

Regarding lysosomal degradation, autophagy (from the Greek, *αυτo-φάγειν* or self-eat) represents three mechanistically distinct intracellular routes for delivering cellular constituents to the lysosome. These include microautophagy, chaperone-mediated autophagy (CMA) and macroautophagy. So far, there is no genetic evidence for the existence of microautophagy or CMA in invertebrates, such as *Caenorhabditis elegans* and *Drosophila melanogaster*. In fact, LAMP2A, a lysosomal glycoprotein that is a crucial factor for CMA in mammals, has no described homolog in yeast, *Drosophila* or *C. elegans* by sequence similarity, suggesting that it is not evolutionarily conserved.

The lysosome is a key organelle, found in all animal cell types, except erythrocytes. Apart from its roles in intracellular signaling and nutrient sensing, it also constitutes the final destination of all autophagic routes, where degradation takes place. Lysosomes contain in their lumen specific lysosomal hydrolases, such as nucleases, lipases, sulfatases, phosphatases and proteases among others [3], that facilitate the degradation of the different types of macromolecules, organelles or pathogens. The proper function of these lysosomal enzymes requires an acidic environment (pH 4.5-5), which is achieved in the lysosomal lumen as a result of an electrochemical gradient maintained mostly by the vacuolar ATPase (v-ATPase) [4], and with the contribution of the CLC-7 chloride channel [5]. The existence of specific v-ATPase inhibitors such as Bafilomycin A1 (BafA1; interacts with the VO ring, inhibiting proton translocation) provides pharmacological tools to block v-ATPase activity and the degradation of the autophagic cargo, thus allowing us to study the autophagic flux [6]. Of note, the v-ATPase exists also in other cellular organelles, such as endosomes, Golgi complex and secretory vesicles [7], but the effects of v-ATPase loss-of-function most severely relate to its role in lysosomal acidification [8].

Microautophagy and CMA do not depend on any organelle other than the lysosome: The former entails the direct engulfment of constituents by an invagination of the lysosomal membrane [9], whereas the latter requires a cytosolic chaperone, Hsc70, which recognizes KFEQR-motif-containing proteins and facilitates their translocation to the lysosomal lumen via an interaction with the lysosomal membrane protein Lamp2A, reviewed in [10]. By contrast, macroautophagy requires the *de novo* biogenesis of a dedicated organelle, the autophagic vesicle (AV) (also known as autophagosome), which selects and delivers the cargo for degradation to the lysosome. This process can be divided in distinct steps, which are well characterized at the molecular level: (1) initiation, (2) elongation/expansion, (3) maturation and finally (4) fusion of the AV with the lysosomes. During the initiation step, biogenesis starts with the nucleation of an isolation membrane, which originates from the endoplasmic reticulum (ER), but potentially also from other intracellular membranes or from the plasma membrane [11]. The isolation membrane expands to a cup-shaped phagophore, which sequesters the cargo, matures and then closes to form the complete, double membrane AV. In the final step, the AV fuses with the lysosome, resulting in the degradation of its inner membrane and luminal cargo. This process requires the orchestrated action of numerous core and auxiliary autophagy proteins, which are evolutionarily conserved from yeast to mammals and have been extensively reviewed previously [12]. Macroautophagy is promoted by AMP activated protein kinase (AMPK), which is a key energy sensor and regulates cellular metabolism to maintain energy homeostasis. Conversely, autophagy is inhibited by the mammalian target of rapamycin (mTOR), a cell-growth regulating hub that integrates growth factor and nutrient signals. At least under conditions of energy depletion or abundance, these kinases exert their effects via distinct phosphorylations on the ULK1 kinase, a major component of the ULK1-complex that is responsible for the initiation of the biogenesis cascade, that enable or inhibit its kinase activity, respectively [13].

In cultured neurons, AV biogenesis is thought to take place continuously in the distal axon, with very few AVs forming in the soma [14, 15]. This spatial arrangement is unique to neurons, as far as we know to date. Retrograde transport of AVs to the soma is facilitated by the interaction of LC3 to the scaffold protein JIP1, which needs to be dephosphorylated. Interestingly, when JIP1 is phosphorylated, as shown with a phosphomimetic JIP1-S421D, autophagosomes aberrantly move anterograde, in the opposite direction [16].

Although macroautophagy was initially considered as a process in bulk (indiscriminate degradation of cellular constituents), it became subsequently clear that a high degree of cargo selectivity is also possible, resulting in the degradation of specific cellular components. For example, selective autophagy has been demonstrated for many organelles and structures: Aggrephagy for aggregates, pexophagy for peroxisomes, mitophagy for mitochondria, ERphagy for the ER, lipophagy for lipids, and myelinophagy for myelin, among others [17]. Selective autophagy relies on the function of selective autophagy receptors (SARs), defined by their ability to associate simultaneously with both the cargo substrate and with components of the autophagic machinery, particularly with the proteins of the Atg8 family (LC3A-C, GABARAPs) and with FIP200 [18]. There is a growing number of selective autophagy pathways and receptors, some of which have been described in neurons, while others have only been characterized in non-neuronal cell types. With the exception of mitophagy which has been studied *in vivo* in the mouse brain under physiological conditions (discussed below), most types of selective autophagy have only been studied in disease paradigms in neurons, where often there is an association with the pathogenesis of different neural disorders reviewed in [18].

## EFFECTS OF NORMAL AGING ON INVERTEBRATE AND MAMMALIAN NERVOUS SYSTEMS

Aging is an unavoidable risk factor for the nervous system and it is linked to the onset of cognitive decline and of different neurodegenerative diseases. Aging is not an acute stress but one that accumulates over time, until a threshold is surpassed, triggering a chain of noticeable effects on cell integrity and function, as a result of compromised basic cellular homeostatic processes. The nervous system is particularly vulnerable to aging because neurons cannot evade death by dividing. In addition, their delicate, highly polarized morphology of axonal and dendritic processes, that underlies their connectivity and function, are easily perturbed by failed homeostasis.

It is well accepted that aging progresses in a coordinated fashion that challenges the physiology of multiple organs. Therefore, aging can compromise brain function both by deteriorating the intrinsic properties of brain cells but also indirectly, by affecting other organs which communicate with the brain. For example, the gut microbiome, which regulates brain function through the gut brain axis (vagus nerve), is also impacted by aging. The negative effects of an aged gut microbiome on the brain has been recently unraveled by a study showing that transplantation of young gut microbiome to the gut of old mice can ameliorate the cognitive deficits of old mice [19, 20]. Another example is inflammation, where systemically circulating pro-inflammatory factors during aging trigger cognitive impairment [21], which can be reversed by young blood transfusion into old mice [22]. Recent work revealed that in aging mice myeloid cell bioenergetics are suppressed in response to increased signaling by the lipid messenger prostaglandin E2 (PGE2), a major modulator of inflammation. Inhibition of myeloid EP2 signaling in aged mice rejuvenated cellular bioenergetics, systemic and brain inflammatory states, hippocampal synaptic plasticity and spatial memory [23].

There are undoubtedly several examples of external cues (including the gut microbiome and inflammation) that can influence brain aging and they have been extensively reviewed elsewhere recently [24]. This review will instead focus on the findings relating to the effects of aging on intrinsic properties of brain cells, including their molecular profile, morphology and function in different invertebrate and mammalian animal models.

### Invertebrate models

Although it is macroscopically evident that *C. elegans* has progressive loss of mobility with aging, as old worms show a declined neuromuscular behavior [25], initial studies using GFP reporters or electron microscopy to visualize neuronal soma and processes, failed to detect any structural decline or cell death in its nervous system with age [26, 27]. These initial results supported that the decline in neuromuscular behavior might be caused by changes in muscle and not in neuronal function. Subsequently, however, of the 302 neurons in the nematode, age-related structural changes have been identified in touch receptor neurons, in cholinergic neurons of the ventral nerve cord [28] and in axons of GABAergic motor neurons in the dorsal and ventral nerve cords, as well as in the nerve ring, the major neuropil of the nematode [29]. Closer examination on touch and GABAergic neurons revealed bubble-like lesions, axon-beading (focal swelling), mishappen neuronal soma, ectopic neurite branching, aberrant neuronal outgrowths, beading and synaptic deterioration, all associated with aging neurons [28–31]. Synaptic deterioration entailed primarily a reduction in the number of synaptic vesicles in nerve ring neurons, which was correlated with decreased locomotion [29] and also supported by the decreased regeneration capacity of motor neurons upon aging [32–35]. Moreover, *C. elegans* shows decreased learning ability upon aging [32, 33, 35]. Notably, neurons displayed a high degree of heterogeneity in their vulnerability to aging and the manifestation of one or more age-related structural deficits.

In the *Drosophila* brain, neurons consist the 85-90% of the total 100.000 brain cells of the organism [36]. Many studies have shown that brains of 50-day-old flies are structurally intact [37–39]. However, upon aging neuronal cells in *Drosophila* brain undergo metabolic changes as shown by decreased levels of ATP in neurons of aged flies (50-day-old) but not in their axons [40] with the use of a fluorescent resonance energy transfer (FRET)-based ATP biosensor [41]. Neurons of aged flies also showed a decrease in their glucose content, in glucose transporter expression and glycolytic enzymes, as well as a decrease in mitochondrial quality [40].

Recently, a single-cell transcriptomic atlas of the aging fly brain was performed [42]. The main conclusion is that while cell identity is retained during aging, there is an exponential decline of gene expression, which is in line with previous findings [43]. There was also an accompanying decrease in cell size with aging and a slight change in cell composition of old brains, as shown by a relative increase of glia, which has also been associated with human brain aging [44]. Finally, genes involved in translation, such as those encoding for ribosomal subunits, were upregulated in the aged brains, whereas genes involved in oxidative phosphorylation were found to be downregulated. Next, the authors used an *in vivo MitoTimer* assay [45] to examine mitochondrial turnover. This assay relies on a time-sensitive fluorescent protein that is targeted to the mitochondrial matrix (*MitoTimer*). Emitted fluorescence of newly translated Timer is green and over time the emission shifts to red. The fluorescent Timer is fused to the mitochondrial targeting sequence of COX8A subunit, to form the *MitoTimer* construct. Using this method, Ferree and colleagues found that mitochondrial turnover decreases in the aged brain, as compared to the young one.

Regarding the behavioral consequences of the aforementioned changes in neuronal profile with aging, memory is one cognitive task that is often thought to decline with aging. One study examined the effects of aging on memory formation, for memories that are associated with survival benefit or not [46]. They found that aged flies of both sexes form robust appetitive memory conditioned with nutritious sugar, which suppresses their high mortality rates during starvation. In contrast, aging impairs the formation of appetitive memory conditioned with non-nutritious sugar that lacks survival benefits for the flies [46]. Moreover, aging enhanced the preference for nutritious sugar over non-nutritious sugar correlated with an age-dependent increase in the expression of neuropeptide F, the fly ortholog of mammalian neuropeptide Y. Furthermore, a subset of dopaminergic neurons that signal the sweet taste of sugar decreases its function with aging, while a subset of dopaminergic neurons that signal the nutritional value of sugar maintains its function with age [46]. Taken together, these results suggest that aging impairs the ability to form memories without survival benefits; however, the ability to form memories with survival benefits is maintained through age-dependent adjustments in the underlying neural circuits.

One recent study examined the transcriptomic profile of fly motor neurons across ages, comparing young (seven days post-eclosion), middle-age (35 days post-eclosion) and aged (45 days post-eclosion) stages and found that the expression of the matrix metalloproteinase 1 (dMMP1) gene reproducibly increased in motor neurons in an age-dependent manner [47]. Modulation of physiological aging also altered the rate of dMMP1 expression, validating dMMP1 expression as a bona fide aging biomarker for fly motor neurons. Temporally controlled overexpression of dMMP1 specifically in motor neurons was sufficient to induce deficits in climbing behavior and cause a decrease in neurotransmitter release at neuromuscular synapses. These deficits were reversible if the dMMP1 expression was shut off again immediately after the onset of motor dysfunction. Therefore, dMMP1 is proposed as a key molecule that contributes to neuronal aging.

In a similar vein, another study examined the effects of aging on neuronal regeneration in the fly [48]. This work focuses on the class IV dendritic arborization (c4da) neuron of the *Drosophila* sensory system, which has a dendritic arbor that undergoes dramatic remodeling during the first three days of adult life and then maintains a relatively stable morphology thereafter. Regeneration is monitored after acute injury and the results indicate that the capacity for regeneration is present in adult neurons but diminishes with aging. Moreover, the authors identified matrix metalloproteinase 2 (Mmp2) as a molecule whose inhibition preserves the extracellular environment characteristics of young adults and leads to increased dendrite regeneration.

**Figure 1:**
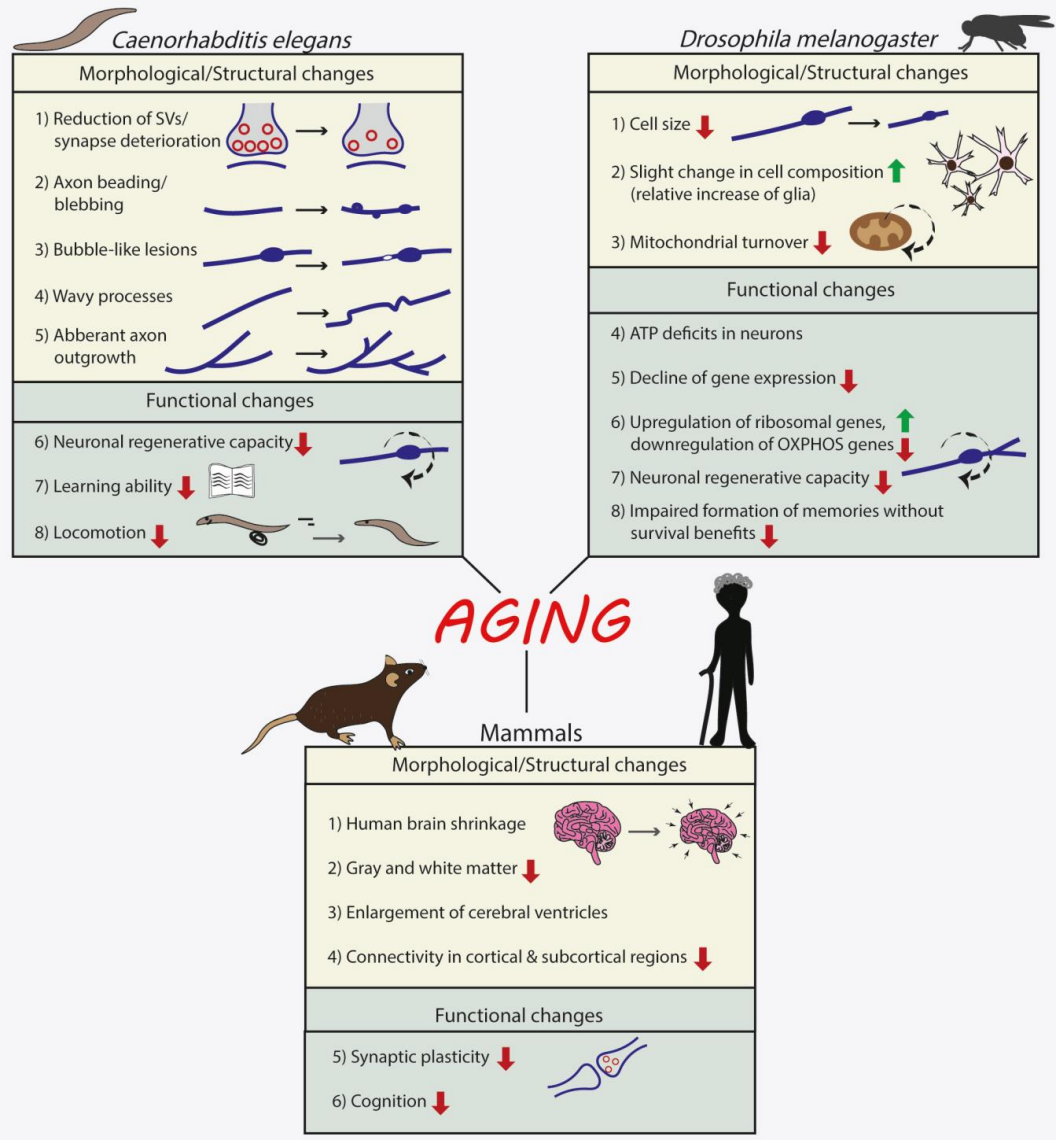
FIGURE 1: Effects of normal aging on the nervous system of invertebrates (*Caenorhabditis elegans and Drosophila melanogaster*) and mammals.

Therefore, using various experimental approaches, both structural and functional deficits have been observed in aged invertebrate neurons, as summarized in **Figure 1**.

### Mammalian models

While invertebrate models have many advantages in studying neurons upon aging, their main limitation is that their nervous system lacks the complexity that characterizes the mammalian brain, at the level of cellular diversity, functional compartmentalization and circuitry. In this section we will overview the key findings on the effects of aging on the brain of humans and of mammalian experimental models, such as the mouse and the rat.

In mammals, aging is tightly associated with a decline in cognitive ability and is considered the main risk factor for developing neurodegenerative diseases. In humans, which are long-lived mammals, it has been long accepted that the human brain shrinks even during normal healthy aging, with reductions in both gray and white matter and an associated enlargement of the cerebral ventricles [49]. Recent imaging advances have helped us to gain more clarity into the changing connectivity and brain structure during aging, both in mice and in humans.

A recent study aimed to decipher the role of healthy aging alone for changes in functional neuronal networks in mice, from developmental adolescence via adulthood to progressing aging, using resting state fMRI at 9.4T [50]. They found that aging is accompanied by a reduced connectivity, which is evident across the brain in cortical and subcortical regions. The reduction of connectivity strength varied between 25% and 70% with most connectivity showing a reduction in strength by approximately 50%. The strongest effect is noted for the rostral dorsal prelimbic cortex with a massive 70% loss in functional connectivity strength at 12–13 months of age. Similar results were also obtained with fMRI datasets from young and aged human subjects [51], which suggested that age has an effect on several whole-brain metrics of functional connectivity.

Another study analyzed multimodal brain imaging data from 21,407 human subjects over the age of 45 from the UK biobank [52]. They found 62 modes of “brain aging” representing different aspects of brain aging, showing distinct patterns of functional and structural brain change. While the individual modes might be related to specific biological processes, the authors further grouped the 62 modes into six coarser ‘mode-clusters', to understand larger patterns of age-related brain changes, and how these relate to other ‘non-imaging' variables such as health parameters and lifestyle. The mode-cluster that was associated with the greatest aging effect, for example, linked cognitive processing speed with brain patterns like ventricular volume and white-matter microstructure. Diabetes, hypertension, and smoking were all risk factors distributed across mode-clusters, suggesting that different aspects of vascular health influence brain aging through different biological processes.

These findings are also well in line with numerous previous studies that collectively highlight how environmental factors influence the rate of brain aging across species. For example, aerobic exercise increases hippocampal volume [53], whereas excessive energy intake and obesity accelerate hippocampal atrophy [54]. Conversely, caloric restriction (CR) and intermittent fasting (IF), which activate autophagy in many tissues, ameliorate structural and functional decline during aging in rodents and monkeys alike [55, 56].

The mouse has been extensively used to characterize the overall changes that occur to the mammalian brain with aging at the molecular level. One study used SILAC-proteomics to measure global changes at the protein level in three mouse brain regions (cortex, hippocampus and cerebellum), comparing adult mice (five months old) with aged (26 months old). Unexpectedly, mean protein abundance changes of more than two-fold between young and old mice were detected in less than 1% of all proteins and very few of these were statistically significant [57]. There were in fact only five proteins which were statistically changed (psap, ctsd, ppt1, tpp1 and asah1), all of which are interestingly lysosomal proteins. Therefore, quite unexpectedly and in contrast to the prevailing view of a deterioration of the neuronal proteome with aging, these results suggest that protein homeostasis remains functional up to a relatively high age.

Another study characterized the effects of aging on the brain proteome by comparing the same three brain regions (cortex, hippocampus and cerebellum) between juvenile (one month old) and middle-aged (twelvemonths old) mice. The highest number of changes was observed in the hippocampus where more than 1760 proteins were affected by aging [58]. One criticism of these findings is that brain maturation is not yet completed in juvenile mice and therefore the observed changes are likely to represent the process of maturation and not of aging.

More recently, Ximerakis and colleagues compared young adult (two to three months old) and old mouse brains (21-23 months old) by single cell transcriptomic analyses. They found that cell identity is largely preserved in old brains and that differences were observed in the variability of transcription between young and old cells in many cell types. However, the directionality of change was not identical among cell types, providing evidence that aging is not broadly associated with increased transcriptional variation in the brain, contrary to what was previously demonstrated for the heart [59].

As summarized in **Figure 1**, also in mammals, aging is accompanied by specific alterations in brain structure and function, and we are starting to gain an important insight into the molecular changes in brain cells that underlie these alterations.

## NEURONAL PROGERIA IN MACROAUTOPHAGY MUTANTS?

### Neuronal survival versus maintenance

The role of autophagy in safeguarding neuronal homeostasis has been investigated by mutations and silencing of autophagy genes in *C. elegans*, and by conditional genetic ablation of key autophagic genes in populations of brain cells, both in *Drosophila* and in mice. In the mouse, conditional ablation of either *atg5* or *atg7* during development, in the progenitors of the entire neural lineage (including all neurons, astrocytes and oligodendrocytes), using the *nestin-cre* deleter, results in neurodegeneration and motor deficits starting at two to three months of age [60, 61]. By contrast, deletion of only one copy of *atg5* or *atg7* in these neural progenitors does not compromise neuronal survival. Therefore, while a reduction in macroautophagy is well tolerated by neurons, its complete ablation leads to neuronal loss, which is accompanied by hallmarks of brain aging, including motor deficits and shorter lifespan.

Recently, human genetic analyses revealed *de novo* mutations in *wdr45*, the gene encoding the autophagy protein WIPI4, in children with neurological phenotypes. The individuals carrying mutations in this gene exhibited static encephalopathy in childhood and neurodegeneration in adulthood [62] and in another case neurodegeneration with brain iron accumulation (BPAN), as well as phenotypes that closely resembled the neurodevelopmental Rett syndrome [63]. Driven by these findings, Zhao and colleagues generated mice with conditional ablation of *wdr45* in the entire neural lineage, under the *nestin-cre* deleter [64]. Confirming the role of WIPI4 in AV biogenesis, these mice exhibited evidence for impaired autophagic flux, including accumulation of p62 and ubiquitin-positive inclusions. However, in contrast to the *nestin-atg5* and *atg7* conditional knockouts, these animals did not show any growth retardation or any behavioral impairments at a young age. Progressively, they developed motor deficits at around twelve months of age and also displayed memory impairment. At the neuronal level, while axonal degeneration and swellings was observed, there was no overt loss of neurons, as in the *atg5* and *atg7* knockouts, possible due to a compensatory role of the three other members of the WIPI family, namely WIPI1, 2 and 3.

Ablation of FIP200, a component of the ULK1 complex which is necessary for the initiation of AV biogenesis, in the neural lineage by the *nestin-cre* deleter, resulted in progressive neuronal loss, spongiosis, axonal and dendritic degeneration in all layers of the cerebellum, including the Purkinje cell layer, starting at the early age of postnatal day 14 (P14) in mice [65]. Spongiosis was also observed in other parts of the brain, suggesting that similar neurodegeneration may also occur in other brain structures. However, staining for the myelin basic protein (MBP) appeared equally robust in the white matter of conditional knockout and control mice, suggesting that at least in the cerebellum myelination was not significantly affected by FIP200 deletion [65].

Atg9 is the only transmembrane autophagy protein, and it is thought to work at the initial stage of AV formation [66]. There are two genes, *Atg9a* and *Atg9b*, that are mammalian orthologs of yeast *atg9*, and the protein products of these genes are functional. ATG9A is ubiquitously expressed in mammalian tissue cells, whereas expression of ATG9B is limited to the placenta and pituitary gland. One study revealed that ATG9A protein is localized throughout neurons including somatodendrites, axons and axonal terminals [67]. Recently, Atg9A was conditionally ablated in the neural lineage by a *nestin-cre* deleter [68], as before. Interestingly, the Atg9A conditional knockouts exhibited more severe phenotypes than *atg7* and *atg5* conditional *nestin-cre* knockouts mice, as about half of them died, within one week after birth and those that survived were much smaller, compared with their floxed control littermates and *atg7-nestin-cre* (atg7-CKO) mice that died at four weeks of age. In addition, these mice experienced severe convulsions, in addition to locomotor ataxia and abnormal limb-clasping reflexes. At the cellular level, neuronal death was observed in Purkinje cells, but not in other areas, and was accompanied by axonal degeneration. Interestingly, the Atg9A conditional knockouts additionally exhibited dysgenesis of the corpus calosum and other white matter tracts, which were not described in the *atg5* and *atg7 nestin*-knockout mice. This was consistent with impaired ability of *atg9a*-deficient cultured neurons to extend neurites, suggesting that the observed phenotypes may entail deficits in neuronal differentiation.

The earliest components of the autophagic machinery to have been conditionally ablated in the neural lineage, using again the *nestin-cre* deleter, are the Ulk1/2 kinases. Ulk1 in particular is a direct target of mTOR and its kinase activity is necessary for activating the ULK1-complex (consisting of Ulk1, Atg13, Atg101 and FIP200) and initiating AV biogenesis. Surprisingly, mice lacking both Ulk1 and Ulk2 in the neural lineage exhibited neither an accumulation of p62 and ubiquitin inclusions, nor any change in the levels of lipidated LC3B-II, which was very different from the *nestin-cre atg5* and *atg7* conditional knockouts [69]. These mice exhibited a progressive degeneration of hippocampal pyramidal neurons, which was attributed to the activation of the unfolded-protein response and a non-canonical role of Ulk1/2 in ER-to-Golgi trafficking.

Related to these findings, the uncoordinated phenotype of *C. elegans* unc-51 (Ulk1/2 homolog) mutants is associated with defective axon guidance [70] but is not recapitulated in other autophagy-deficient mutants such as *epg-1* (*Atg13*) and *epg-9* (*Atg101*) mutants [71, 72]. RNAi-mediated silencing of other autophagy-related genes, e.g., *bec-1 (Atg6), M7.5 (Atg7), lgg-1 (Atg8),* or *F41E6.5 (Atg18)*, also does not replicate the neuronal defects observed in unc-51 mutants [70]. Indeed, the role of ULK/ATG1 in ER-to-Golgi trafficking may be conserved in *C. elegans* because unc-51 mutants also exhibit abnormal COPII assembly and trafficking of MOD-5 (SERT), inviting the speculation that their uncoordinated phenotype may be also attributed to an unconventional function of unc-51 in ER-to-Golgi trafficking.

In *Drosophila*, autophagy gene null mutants showed that loss of *atg7* leads to the accumulation of non-degraded proteins, premature neuronal aging and apoptosis, ataxia and short lifespan [73]. Several other components of the autophagic machinery including FIP200 [74], Atg8a [75], Atg9 [76], and Atg16 [77] were also found to be essential for the maintenance of proper neural functions and normal lifespan. However, as all brain cells are affected, it's not possible to determine from these mouse and *Drosophila* studies whether autophagy is required cell autonomously in neurons for their survival or if neuronal loss is the indirect result of perturbed homeostasis in other brain cells, such as glia.

A more recent study examined neuronal survival after ablation of *atg5* in pallial progenitors in the dorsal telencephalon, which give rise to excitatory glutamatergic neurons and subsequently to astrocytes, using the *emx1-cre* deleter in the mouse [78]. In line with previous studies, neuronal loss was observed in the forebrain of these conditional knockouts at four months of age, but not earlier. In this case also, ablation affects both glutamatergic neurons and astrocytes, something that has not been discussed in this paper, and more work is needed to discriminate the cell-autonomous requirements of autophagy in these two different cell populations.

Another study examined mice with ablation of *atg7* by a *camK2a-cre* deleter, which drives expression specifically in mature excitatory neurons in the forebrain starting at around postnatal day 14-20 [79]. Although the authors did not directly examine neuronal survival and did not report any overt loss of neurons, they indicated that unlike the *nestin-Atg5* and *nestin-Atg7* conditional knockouts, the *camk2a-atg7* knockout mice did not exhibit motor deficits. Therefore, it still remains unclear whether excitatory glutamatergic neurons, which constitute the majority of neurons in the mammalian forebrain, require autophagy cell-autonomously in order to survive. Moreover, it's also elusive whether the developmental stage is also important, as studies in embryonic stages show more severe phenotypes compared to those in postnatal timepoints, when neurons are already postmitotic and fully differentiated.

One neuronal population which is clearly susceptible to perturbation of macroautophagy are the Purkinje cells of the cerebellum. These cells are GABAergic inhibitory projection neurons and constitute the only output of the cerebellum, with major roles in motor learning among other tasks. Interestingly, ablation of *atg7* in postmitotic Purkinje cells, using a *pcp2-cre* deleter, resulted in a cell-autonomous dystrophy as well as axon terminal swelling and degeneration, but no differences in the Purkinje dendritic tree or spine morphology [80]. These findings invite the speculation that the motor deficits observed in the aforementioned *nestin-atg5* [60] and *nestin-atg7* [61] conditional knockout mice may be attributed to the loss of Purkinje cells. Another neuronal population which appears vulnerable to autophagy deficiency are the rods in the mouse retina. Ablation of *atg5* in rod photoreceptors led to their progressive degeneration beginning at P56 such that by ten months old only very few rods remained [81].

By contrast, some neuronal populations seem unsusceptible to loss of autophagy for their survival. Kaushik and colleagues conditionally ablated *atg7* in the AgRP hypothalamic neurons, using the *agrp-cre* deleter mouse [82]. These neurons, together with the POMC (proopiomelanocortin) hypothalamic neurons, form a focal point for the integration of nutritional and metabolic cues, central and peripheral neural afferents [83], and action of adiposity hormones such as leptin and insulin [84]. They found that while autophagy deficient AgRP (Agouti-related protein) neurons do not exhibit higher mortality, they result in significantly reduced body weight, total fat mass and white adipose tissue (WAT) mass, without affecting food intake. The same group generated mice with conditional ablation of *atg7* in the neighboring POMC-positive hypothalamic neurons [85] using a *pomc-cre* deleter and they similarly found no increased apoptosis. Similarly, conditional ablation of *atg7* in dopaminergic neurons using a *dat-cre* deleter did not cause neurodegeneration [86]. Therefore, autophagy deficiency in the dopamine system and in the hypothalamus, in two distinct hypothalamic neuronal populations, did not compromise neuronal survival, further pointing to the varying effects of autophagy perturbation depending on the neuronal cellular context.

The analyses of the different conditional autophagy knockouts, which are summarized in **Table 1**, clearly suggest that some neuronal populations, such as the Purkinje neurons, are particularly vulnerable to autophagy deficiency and undergo acute death soon after the onset of the deficiency, while others, such as hypothalamic neurons, are unaffected by chronic autophagy deficiency, at least in terms of their survival. It also appears, however, that for some populations, such as the majority excitatory glutamatergic neurons, the effects of autophagy deficiency may largely depend on the maturation stage of the onset of the deficiency, as well as its duration. More work is needed to distinguish between the possible roles of autophagy in acute neuronal survival *per se* versus in neuronal maintenance, which is more cumulative and more related to aging.

**Table 1.**
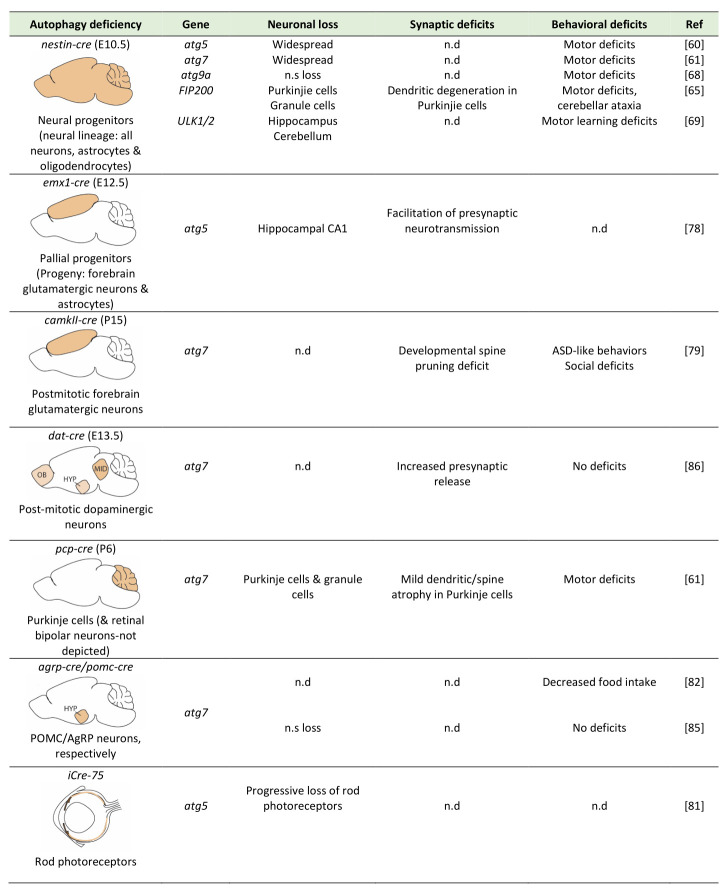
Summary of the effects of conditional ablation of autophagy genes in different brain cells on neuronal survival, synapses and behaviors. The first column provides a schematic representation of the site of autophagy-gene ablation (in beige colour), of the Cre-deleter used, the onset of ablation (in parentheses) and the brain cells affected.

Abbreviations: n.d: not determined, n.s: not significant, CA1: cornu ammonis 1, POMC: Pro-opiomelanocortin, AgRP: Agouti-related protein, OB: Olfactory bulbs, HYP: Hypothalamus, Mid: Midbrain.

### Impaired synapse in autophagy mutants

The synaptic functions of autophagy have been previously reviewed in depth elsewhere [87, 88]. Briefly, in the past few years, several studies have demonstrated that in neurons the autophagic machinery has adapted to serve functions that pertain specifically to the synapse. Hernandez and colleagues showed that autophagy-deficient dopaminergic neurons survive well, as mentioned earlier, however, they exhibit presynaptic deficits. Chronic macroautophagy deficiency in dopamine neurons resulted in increased size of axon profiles, increased evoked dopamine release, and more rapid presynaptic recovery in eight weeks old mice [86]. Moreover, in mice with intact macroautophagy, mTOR inhibition with rapamycin acutely increased AV formation in axons, decreased the number of synaptic vesicles, and depressed evoked dopamine release, but these effects were not observed in dopamine neuron-specific macroautophagy-deficient mice. Taken together, these findings suggest that mTOR-dependent local axonal macroautophagy can rapidly regulate presynaptic structure and function, potentially by regulating the turnover and size of the synaptic vesicle pool.

In line with this, a more recent study found that autophagy-deficient excitatory glutamatergic neurons also exhibit presynaptic defects [78]. In this case, as mentioned earlier, *atg5* was ablated in pallial progenitors using an *Emx1-Cre* deleter mouse, resulting in autophagy deficiency in all forebrain glutamatergic neurons and astrocytes (**Table 1**). The authors do not comment on the fact that astrocytes are also affected. Field recordings in hippocampal slice preparations as well as in autophagy-deficient cultured neurons indicated that loss of neuronal autophagy causes cell-autonomous facilitation of presynaptic neurotransmission that is not explained by alterations in the number or density of synapses, the excitatory versus inhibitory synapse ratio, presynaptic vesicle numbers, pool sizes or synaptic vesicle? localization. Using cultured neurons, they propose instead, that the presynaptic phenotype is attributed to the accumulation of axonal tubular ER, resulting to elevated calcium release from internal stores.

By contrast, accumulating evidence supports the notion that the synaptic vesicle pool is regulated by autophagic degradation and several active zone proteins have been demonstrated to participate in this process. In *C. elegans*, the synaptic vesicle kinesin-like protein UNC-104/KIF1A was shown to transport ATG-9 to presynaptic sites of AIY interneurons. This mechanism was suggested to support the local ATG9-mediated synaptic AV formation and its role in synaptic vesicle clustering and turnover and active zone assembly in invertebrate neurons [89].

In line with this, a recent study showed that loss of Piccolo and Basoon, two key scaffolding proteins of the presynaptic active zone, results in increased number of presynaptic AVs and degradation of synaptic vesicles [90]. Moreover, they show that the CC2 region of Bassoon directly binds Atg5 and possibly inhibits Atg5-Atg12 complex formation and initiation of presynaptic autophagy. Interestingly, a recent study showed that the E3 ligase Parkin is required for increased autophagy in Bassoon-deficient neurons as the knockdown of Parkin normalized autophagy and synaptic vesicle protein levels and rescued impaired synaptic vesicle recycling [91]. A direct link between synaptic vesicles and autophagy was also identified [92], whereby the small GTPase Rab26 is enriched on synaptic vesicles and binds to ATG16L in its activated GTP-bound form. This interaction was shown to recruit the autophagy initiation machinery directly to synaptic vesicles and activation of Rab26 was later shown to be triggered by the guanine exchanged factor Plekhg5 [93].

Synaptic functions of autophagy have also been suggested in postsynaptic compartments, mainly dendrites. For example, an earlier study by Shehata and colleagues suggested that AVs are enriched in dendrites and degrade glutamate receptors, following treatment with a brief pulse of N-methyl-D-aspartate (NMDA), an agonist of NMDARs, which is known to induce in culture the cascade leading to NMDAR-dependent long-term depression [94]. This is a form of plasticity that entails the removal of glutamate α-amino-3-hydroxy-5-methyl-4-isoxazolepropionic acid (AMPA) receptors from the postsynaptic membrane and their subsequent degradation is involved in key behaviors, such as memory erasure and cognitive flexibility. In a second study, Shehata and colleagues also demonstrated that autophagy is required for memory erasure via the destabilization of postsynaptic structures [95]. In line with these findings, our recent work further demonstrates that autophagy is required for the two major forms of long-term depression? (LTD), facilitated by activation of either NMDA or Group1 metabotropic glutamate receptors, and that during this process it degrades AMPA receptors but also key scaffold molecules of the postsynapse, such as PSD95 [96, 97]. These findings are also in agreement with previous work, showing that ablation of *atg7* in postmitotic excitatory glutamatergic neurons, using the *camK2a-cre* deleter in mice, prevents the developmental pruning of postsynaptic structures (known as dendritic spines), a process that is known to be facilitated by LTD-like mechanisms [79]. In a similar vein, our previous findings also demonstrated that uncontrolled upregulation of neuronal autophagy, as it is the case in mice with conditional deletion of brain-derived neurotrophic factor (BDNF), results in the degradation of postsynaptic molecules, interfering with the ability of neurons to undergo synaptic plasticity [98]. However, one recent study showed conflicting results, showing that suppression of autophagy facilitates LTD [99]. More work is needed to understand if the contradictory results could be explained by some experiments being performed at different postnatal ages, or by the fact that pharmacological agents used in the latter study have pleiotropic effects in addition to modulating autophagy.

Recently, it was also shown that genetically impairing autophagy, by RNAi silencing of *atg5* or *atg9* under the pan-neuronal driver elav-Gal4, within the major learning-center of *Drosophila*, the mushroom body (MB), sufficed to trigger brain-wide deficits in presynaptic organization in a non-cell autonomous manner, and a decline in aversive olfactory memory, a hallmark of the aging process. In contrast, attenuating autophagy in other brain centers had no effect. This work further identified the metabolism-related NPY-type neuropeptide within the MBs as a regulator of premature metaplasticity and consequently a decay of memory formation capability. Taken together, these results provide evidence that the autophagy status of the MB, the fly brain integration center, is important in tuning the information processing of an entire brain [100].

In summary, there is accumulating evidence for specific roles of autophagy both in presynaptic and postsynaptic compartments. Moreover, key molecules are identified that facilitate unique interactions and cross-regulation between the autophagic and synaptic machineries.

## EFFECTS OF NORMAL AGING ON NEURONAL MACROAUTOPHAGY IN INVERTEBRATES AND MAMMALS

**Figure 2:**
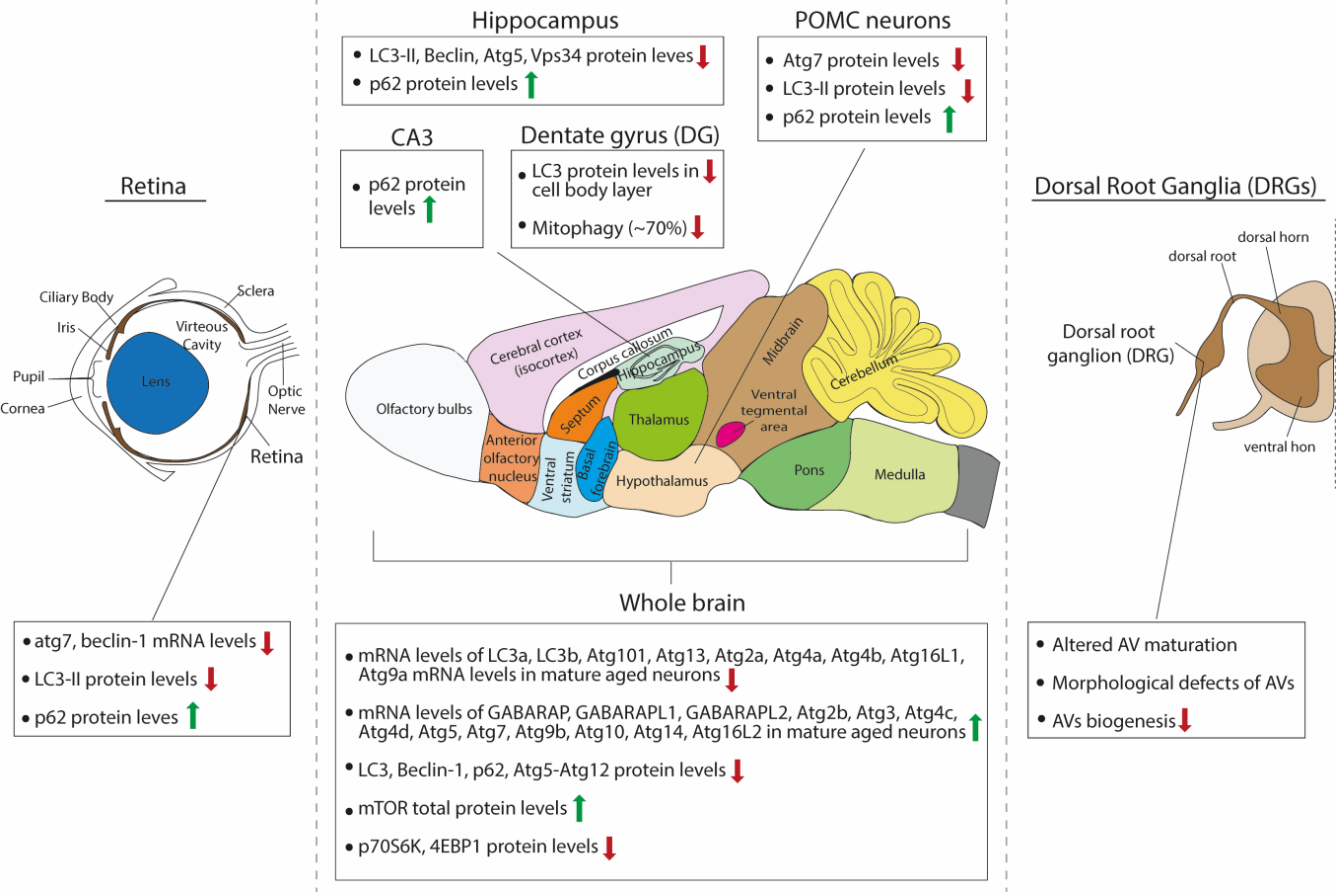
FIGURE 2: Schematic summary of the observed effects of aging on macroautophagy in the rodent brain.

The effects of aging on neuronal macroautophagy have been investigated in two well studied invertebrates, *C. elegans* [101] and *Drosophila*, as well as in mammalian models. Each model offers unique advantages, allowing a variety of experimental approaches in studying autophagy upon neuronal aging. In this section, we comparatively overview the main findings of how the autophagic machinery is perturbed in the nervous systems of different model organisms during normal aging (also summarized in **Figure 2**).

### Effects of aging on neuronal autophagy in invertebrates

As mentioned earlier, macro-autophagy is the only type of autophagy which is highly conserved in evolution and has been extensively studied in invertebrate models under physiological conditions as well as upon aging.

Both *C. elegans* and *Drosophila*, have relatively short lifespans, of 18 and 70 days, respectively [102]. Therefore, they are well-suited models for studying mechanisms of longevity and in particular the role of macroautophagy in organismal lifespan. In *C. elegans*, it is noteworthy that all genetic interventions that increase lifespan result in autophagy induction and require autophagy for the observed longevity [103]. Similarly, in *Drosophila*, loss-of-function mutations of *atg7* and *atg8* genes reduce lifespan [73]. Moderate genetic or pharmacological induction of autophagy results in lifespan extension, although a strong induction may also have deleterious effects [104]. These findings have been extensively reviewed elsewhere [105, 106]. Here, we will focus on the findings relating to neuronal autophagy in aging studies of these models [107].

In *C. elegans,* macroautophagy and its flux to the lysosome have been mainly monitored in living animals by overexpression of LGG-1, the worm ortholog of *Atg8* genes, fused at its N-terminus to one fluorophore (GFP or mCherry), or to a tandem of mCherry and GFP. In both cases, fluorescent LGG-1 appears punctate when associated with the autophagic membranes, either at the stage of the phagophore or the complete AV. The GFP-LGG-1 construct reports on the number or pool of phagophores and AVs, as the GFP is quenched by the low pH or the autolysosome. By contrast, the tandem mCherry-GFP-LGG-1 construct allows for the visualization of phagophores and AVs as yellow puncta but also of acidic amphisomes and autolysosomes as red puncta, since mCherry still fluoresces in acidic environments. Therefore, the latter reporter is used for measuring the autophagic flux, as a ratio of yellow and red structures.

In one study, the mCherry::LGG-1 construct was expressed in dopaminergic neurons under the dat-1 promoter and the mCherry-positive puncta were counted in different ages and normalized to GFP expressed from the same promoter [108]. These experiments indicated that there is a significant increase in the mCherry signal with increasing age. However, as this construct does not report on the autophagic flux, but only on the steady state pool of LGG1-positive structures, it is not possible to conclude whether this increase represents an upregulation or a deficit in autophagic activity.

Using the tandem mCherry-GFP-LGG-1 reporter, Chang and colleagues reported that the autophagic flux in *C. elegans* decreases with age in the nerve-ring neurons, among other tissues, in a spatiotemporal manner [109]. In particular, they found that the number of green LGG1 puncta, reporting AVs and phagophores, is significantly increased in aged (seven and ten days post larva) compared to young (one day post larva) worms. However, the number of red-only puncta, reporting autolysosomes, was significantly decreased with aging, suggesting an age-associated deficit in autophagic flux. Given the accumulation of the green puncta in the aged neurons, these findings invite the speculation that the problem may not lie in the biogenesis of AVs, but rather in their final degradation in the lysosome, which may be attributed to impaired maturation of AVs and their ability to fuse to lysosomes, or alternatively to problems in lysosomal function.

In another study, a new assay was developed, whereby the *C. elegans* LGG-1 was tagged with two fluorescent proteins connected by a flexible, protease-sensitive linker (dFP: dual Fluorescent Protein tag). Fusion of the AV to the lysosome exposes LGG-1 to the lysosomal proteases, which cleave the linker between the fluorescent tags, releasing monomeric FP (mFP), which is tightly folded and resistant to proteases. Therefore, autophagic flux is measured as the ratio of dFP and mFP [110]. This assay was validated under conditions of starvation, the best described inducer of autophagy, as well as under conditions where lysosomal activity was blocked with Chloroquine, a drug that interferes with lysosome acidification. To measure autophagy in different tissues, strains were constructed with dFP::LGG-1 expression under the control of tissue-specific promoters, including the neuron-specific promoter Rab3p. In contrast to previous findings, this study concluded that aging is accompanied by an increase in the autophagic flux in neurons, as well as other tissues [110].

Early work in *C. elegans* also examined the effects of aging on lysosomes, the endpoint organelle of the autophagic flux. Sarkis and colleagues examined the lysosomal protease activity during aging in *C. elegans* (day three to day eleven) and found that the activity of the lysosomal proteases cathepsin D, Ce1 and Ce2 declined with age but the activity of cathepsin CeX was unchanged [111]. These data support but do not prove the hypothesis that declined lysosomal activity in older animals may lead also to a reduced protein turnover rate [111]. On the other hand, not all the lysosomal proteases decline with age as the activity of lysosomal glycosidases and acid phosphatases in *C. elegans* was strongly increased in aged animals, showing that different enzymes of the lysosome are not affected similarly as the animal ages [112].

Therefore, on the one hand it's undisputable that reduced autophagy shortens the lifespan of *C. elegans* and that in some cases reinstating autophagy in neurons is sufficient to ameliorate lifespan reduction. On the other hand, the regulation of the autophagic activity in different neurons of the nematode during aging remains inconclusive, as studies using different assays have reported conflicting results.

Increased levels of the homolog of p62 (Ref(2)P) as well as ubiquitinated neural protein aggregates has been reported in aged wild-type *Drosophila* as well as in autophagy mutants of the fly [113, 114], suggesting that in the fly brain there may also be an age-related decline in autophagic activity. An early study by Simonsen and colleagues demonstrated that macroautophagy maintenance in the nervous system of adult flies facilitates lifespan extension and reduces accumulation of ubiquitinated proteins, a hallmark of cellular aging [115]. Upon aging, the expression levels of several autophagy genes, including *atg2, atg8α, atg18* and blue cheese or *bchs* gene, (homologue of human ALFY) were found to be reduced in the fly brain as determined by quantitative real-time PCR (qRT-PCR) experiments comparing young (one day old) and adult and aged (ranging from three to seven weeks old) flies [115], whereas *atg1* and *atg5* were unchanged. In addition, the protein levels of Atg8, both the cytosolic Atg8-I and lapidated Atg8-II, were reduced in aged (four weeks old) compared to one day old fly brains. It is noteworthy that the comparison was always made to the one day old fly brain, which is immature and not yet adult, hence it's not clear whether a significant decrease is indeed observed between adult and aged brain for all transcripts tested as well as for Atg8 protein. At the same time, the *rpn6* transcript, encoding a proteasomal subunit protein, was found to be upregulated in the aged brain, possibly suggesting a putative upregulation of proteasomal activity to compensate an age-related deficit in autophagy [75]. In line with impaired clearance of proteins, a significant accumulation of ubiquitinated proteins was observed in the brain starting from four weeks of age. In summary, this study provides some evidence for reduced expression of some autophagy proteins in the aged fly brain, however, it does not provide any direct measurement of reduced autophagic activity in aged fly neurons.

More recent findings also support the age-related decline in autophagy in the *Drosophila* brain. Omata and colleagues have shown that the expression of *atg8a* and *atg18* is significantly downregulated in the fly head in an age-dependent manner (four days young vs 30 days old heads), as well as that of *atg1*, which was found unchanged in the previous study [75]. This work further found that reduced autophagy activity correlated with late-onset neuronal dysfunction caused by neuronal induction of amyloid β (Aβ), supporting the idea that age-related dysfunction of autophagy is a causative element in onset and progression of Alzheimer's disease (AD) [116].

Another study focused on the role of polyamines in regulating brain autophagy in aging. This work reported that levels of polyamines (spermidine and putrescine) decreased in aging fruit flies, correlating with declining memory abilities. Spermidine feeding restored juvenile polyamine levels and specifically ameliorated age-induced memory without affecting other behaviors that decline with aging, such as odor avoidance. Spermidine feeding enhanced autophagy and required autophagy for its effects on memory, as these effects were abrogated in *atg7* and *atg8a* mutant flies. Although this work does not directly identify neurons as the cellular substrates for the direct action of spermidine, it does implicate autophagy in the memory-boosting effects of spermidine in aged flies [117].

In addition to studying general autophagy, a recent study generated flies expressing the mito-Keima construct in order to monitor mitophagy *in vivo*. The fluorescent protein Keima, which is tagged with a mitochondrial targeting sequence, has an excitation spectrum that changes according to pH. A short wavelength (440 nm) is predominant for excitation in a neutral environment, whereas a long wavelength (586 nm) is predominant in an acidic environment. Using live mito-Keima imaging and correlative light and electron microscopy (CLEM), the authors showed that mitophagy occurs in muscle cells and in dopaminergic neurons *in vivo*, under basal conditions, in the absence of any exogenous mitochondrial toxins. Moreover, they showed that this selective form of autophagy increases with aging in the fly brain, which is in contrast to the general consensus that autophagic activity declines with aging. This age-related increase in mitophagy can be ameliorated by loss of Pink1 or Parkin, two proteins which form the core machinery for the recognition of mitochondria by autophagy and which are often mutated in Parkinson's disease patients [118].

### Effects of aging on brain autophagy in mammals

#### Whole brain

The same single-cell brain transcriptomic study by Ximerakis and colleagues [119], discussed above, interestingly reported that the transcripts of nine core autophagy genes were found to be downregulated in aged neurons (*lc3a, lc3b, atg101, atg13, atg4a, atg4b, atg2a, atg16l1* and *atg9a*), while the transcripts of thirteen core autophagy genes were upregulated in aged neurons (*gabarap, gabarapl1, gabarapl2, atg3, atg4d, atg10, atg14, atg16L2, atg4c, atg5, atg9b, atg2b* and *atg7*), as compared to adult. Moreover, the direction of change for some of these genes differed between neurons and other brain cells types, such as oligodendrocytes. These findings are interesting for two reasons: First, they raise the issue of cellular heterogeneity in the brain and invite the speculation that aging may differentially impact on autophagy in the different neuronal or glial populations, even within the same brain structure or region. Second, as very little is still known on the distinct or overlapping functions of different Atg8 proteins in selective autophagy, they remind us that LC3B, which is often studied, is only one of the six Atg8-family proteins that are involved in autophagy. Most of the studies that are reviewed below base their conclusion on autophagic flux changes on the levels of LC3B, however, this study suggests that while LC3B levels may decrease with aging, the levels of other Atg8 proteins may increase and may in fact have compensatory functions.

Based on western blot analyses with antibodies against LC3B, Beclin-1, p62 and atg5-atg12, several studies have independently described an aging-associated reduction of neuronal macroautophagy in the mouse brain. In one study, Ott and colleagues compared the levels of some autophagy proteins in whole brain lysates of young adult (two months old), adult (six months old) and aged (18-25 months old) mice [120]. They found that p62 and Atg5-Atg12 levels were not significantly changed between ages, whereas Beclin1 levels were reduced in adult and aged brain compared to young adult, but were similar between adult and aged conditions. Additionally, the total levels of mTOR were significantly increased in the aged brain compared to young adult, but not compared to adult. The levels of mTOR downstream effectors p70S6K and 4E-BP1 were significantly decreased in the aged brain compared to young adult, but not compared to adult and the ratios of p-p70S6k/p70S6K and p-4E-BP1/4E-BP1 were unchanged between ages. Although this study claims that there is an age-associated impairment of brain autophagy, this cannot be concluded from these data, as there were no differences between adult and aged brains. Therefore, one cannot exclude the possibility that differences observed between young and aged brains entail the factor of maturation.

Another study compared p62 and LC3B-I and -II protein levels by Western blot analysis, in the cortex, hippocampus, midbrain and cerebellum of adult (three months old) and aged (24 months old) mice [121]. They found that these proteins are all increased in the aged brain across regions, although these results were not quantified. Moreover, they found an accompanying increase in the levels of BAG3 and a decrease in the levels of BAG1 across the aged brain, two proteins belonging to the Bcl-2-associated athanogen family [122]. They showed that BAG1 and BAG3 act positive regulators of the ubiquitin proteasome and autophagic pathway, respectively, and hence propose that autophagy is induced in the aged brain to compensate for reduced proteasomal activity.

Naked-mole rats are the longest-lived rodents and are known to have higher basal autophagy levels compared to mice, rats and other mammals [123, 124]. A recent study demonstrated that Beclin1 levels progressively decrease with aging and are significantly lower in the oldest age group, whereas the LC3B-II/LC3B-I ratio remains constant with age [124]. At the same time, the PI3K and Akt pathway activity both decrease in the oldest age group, as well as the p-mTOR/mTOR ratio. The conclusion of this work is that the naked mole rat brain maintains high levels of autophagic activity until the oldest age, which may be functionally related to its longevity.

Accumulating evidence suggests that in addition to challenging the autophagic machinery, aging may also compromise different aspects of lysosomal biology in neurons. Several studies have reported aging-related changes in the activities of Cathepsins and an overall decline in lysosomal hydrolysis upon aging in various tissues, including the brain [125]. This is also in agreement with an age-dependent accumulation of lipofuscin, most evident in neurons, and the enlargement of the lysosomes, both considered indications of slowed substrate clearance. Lipofuscin is the product of iron-catalyzed oxidative modifications of macromolecules that consist aggregates and are destined for degradation to the lysosome. This auto-fluorescent pigment consists indigestible material that behaves as a hub for lysosomal enzymes to gather for its digestion [126]. This fatally leads to a decrease in the pool of functional lysosomes as most lysosomal enzymes end up in the lipofuscin-positive lysosomes to help digestion rather than in active lysosomes, driving lysosomal activity to be diminished. Moreover, decreased activity of the v-ATPase has been associated to age-related decrease in lysosomal function and neurodegenerative diseases [127–132]. In addition, it has been proposed that aging may also trigger lysosomal membrane permeabilization (LMP), whereby the integrity of the lysosomal membrane is compromised and allows the translocation of the luminal contents to the cytoplasm. This in turn, is thought to trigger a so-called lysosome-dependent cell death. This process has been reviewed elsewhere in great detail [133–136].

#### Hypothalamus

As previously described, Kaushik and colleagues investigated the role of macroautophagy within the POMC neurons of the mouse, a specific subpopulation of hypothalamic neurons that integrate nutritional and hormonal cues to control energy balance [85]. POMC neurons express POMC preproprotein that is processed to generate adrenocorticotrophic hormone (ACTH) and α-melanocyte-stimulating hormone (MSH). α-MSH activates central melanocortin receptors to curtail food intake and promote energy expenditure by modulating sympathetic outputs to the periphery. Autophagy deficiency in POMC neurons in adult mice resulted in decreased MSH levels, adiposity and impaired lipolysis as well as altered glucose homeostasis. Interestingly, aged mice (22 months old) presented the same phenotypes and exhibited decreased macroautophagy, as assessed by decreased Atg7 and LC3B-II protein levels, and increased p62 levels [85]. Therefore, these findings suggest that there is an overall reduction in autophagic activity in aged hypothalamic neurons.

#### Retina

As retinopathies are very common upon aging and can lead to blindness, one study investigated the role of autophagy in the mouse retina during aging [137]. The authors compared the mRNA levels of Atg7 and beclin1 between young adult (three months old), adult (twelve months old) and aged (22 months old) retinas and found that both were significantly reduced in adult compared to young adult mice. However, no further reduction was observed between adult and aged mice. They also calculated the protein levels of LC3B-II in the absence and presence of a lysosomal inhibitor. As expected, LC3B-II levels significantly increased in the presence of the inhibitor in young adult retinas but failed to increase in the adult and aged retina, suggesting that less LC3-II is fluxed through the lysosome. In line with this, p62 levels were increased in adult retina compared to young adult, but no further accumulation was observed in the aged retina. In parallel, the adult and aged retina exhibited increased levels of Lamp2A, a lysosomal protein that also participates in CMA, compared to the young adult. Whether this increase represents increased CMA activity is unclear, as the authors investigated CMA activity only in cultured cell lines but not in retinas at the different ages [137].

#### Hippocampus

The hippocampus is a well-studied brain structure in the field of aging, due to its essential role in memory formation and learning, cognitive functions that deteriorate with age. A recent study investigated how autophagy changes in the hippocampus of mice with aging [138]. Here, the authors compared three months and 16 months old mice and found that steady state levels of LC3B-II, Beclin 1, Atg5 and Vsp34 that all decrease in hippocampal lysates of the older animals. This is accompanied by an accumulation of p62-positive puncta, suggesting either decreased autophagic flux in general or at least decreased aggrephagy, since p62 is mainly an autophagy receptor for aggregates. Moreover, the performance of the 16 months old mice in two memory tests, the novel object recognition and the contextual fear conditioning paradigms, was reduced compared to the three months old mice, and these deficits were partially rescued by intra-hippocampal injection of a cell-permeable TAT-beclin-1 peptide, which was previously shown to efficiently increase autophagy [138]. Taken together, these results suggest that boosting autophagy in the hippocampus is beneficial for partially restoring age-related decline in memory performance, while the cellular types involved remain elusive, as TAT-Beclin-1 is indiscriminately incorporated in all different types of neurons and glia in the hippocampus. Interestingly, this study went on to identify the hormone osteocalcin, a bone-derived molecule, as a direct hormonal inducer of hippocampal autophagy. Similarly, another study also showed that autophagy levels are decreased in mossy fiber synapses onto CA3 pyramidal neurons that are formed by hippocampal granule cells. This was demonstrated by decreased levels of LC3 and increased levels of p62 in the aged (24 months old) mouse dentate gyrus (DG) and CA3 area, when compared to young (five months old) animals [139].

While most work has been focused on assessing changes in baseline autophagy in general, one study investigated the effects of aging on mitophagy *in vivo*. To this end, Sun and colleagues generated a transgenic mouse model expressing a mito-Keima. [140]. In this study, the mito-Keima fluorescence signal from 561-nm laser excitation (acidic) was depicted in red and the signal from 458-nm laser excitation (neutral pH) in green and the level of mitophagy was measured as the total number of red pixels divided by the total number of all pixels. Interestingly, within the brain there was considerable heterogeneity with regards to baseline mitophagy levels. The cortex, striatum, and substantia nigra had seemingly modest levels of basal mitophagy, whereas mitophagy was enhanced in the DG and the lateral ventricle, areas known to be enriched for adult neurogenesis. The Purkinje cell layer within the cerebellum had a similarly high level of mitophagy. This was also confirmed by another study, which monitored mitophagy *in vivo* with a Mito-QC transgenic mouse, expressing a mCherry-GFP-FIS1 construct, and similarly found high levels of mitophagy in Purkinje neurons [141]. When *atg5* was conditionally deleted in the entire mouse brain lineage using *nestin-cre* deleter, there was a marked diminution of approximately 80%, but not complete absence, of the mito-Keima red signal in regions with increased rates of mitophagy such as the DG and Purkinje cell area of the cerebellum. The authors speculate that the remaining 20% of mitophagy presumably represents the degree of ATG5/LC-3-independent mitophagy that occurs within this tissue. They next sought to characterize whether normal physiological aging impacts mitophagy in the brain and focused their analysis on the DG region because of its higher levels of basal mitophagy and its importance in memory and learning. They found that while in three months old mice the DG area demonstrated a high level of mitophagy, in older mice (21 months old) there was a marked reduction by approximately 70%. It cannot be excluded however, that some of this reduction comes from changes in the cellular composition of the DG, as in particular the number of neural stem cells is known to decrease with age in this area [142].

#### Peripheral neurons

Recent work demonstrated that in peripheral neurons aging is accompanied by altered maturation of AVs. Taking advantage of the fact that unlike central nervous system (CNS) neurons, peripheral neurons can be cultured from postnatal and adult ages, the authors cultured dissociated dorsal root ganglia (DRG) neurons, which were isolated from young (one month old) or aged (17 months old) mice. After over-expression of a GFP-LC3B construct, they identified AV biogenesis events as the formation of discrete GFP-LC3B puncta in axon tips, visible above the background cytoplasmic GFP-LC3B signal. Strikingly, the rate of AV biogenesis significantly decreased in the aged neurons by 53%, as compared to the young controls. In addition, by electron microscopy analysis, aged peripheral neurons exhibited morphologically aberrant structures with a multilamellar (onion skin-like) appearances, suggesting that they may be malformed AVs. However, no immune electron microscopy experiments were performed to associate these aberrant structures with any autophagy markers, thus it remains unclear if these structures are indeed autophagic in nature. This paper suggests that these are stalled AVs, which accumulate phagophore markers, such as WIPI2B, but fail to recruit LC3B [143]. They propose that isolation membrane formation needs the unphosphorylated form of WIPI2B but this form needs to be phosphorylated back in order to aid the expansion of the isolation membrane. The aforementioned study suggests the phosphorylation state of WIPI2B to be a key event in producing the stalled AVs in aged peripheral neurons and propose WIPI2B as a therapeutic target in neurodegeneration during aging [143, 144].

## CONCLUDING REMARKS

Undoubtedly, great advancement has been achieved in the recent years in understanding the intricate interplay between macroautophagy and normal aging in the nervous system. This progress is of paramount importance, as the size of the aged human population rises constantly and maintaining this population healthy and functional has direct implications for the individuals and for the society [145, 146]. Macroautophagy is a well-characterized cellular homeostatic process, which is linked to several age-related neurodegenerative disorders, reviewed extensively in [128, 147, 148], and therefore of great interest also in the context of normal aging. As macroautophagy is regulated by various lifestyle and environmental factors, including the amount and quality of food intake, exercise and stress, among others, it constitutes a key target for achieving proper homeostasis as adults and maintaining it in the aged brain.

While it appears that the literature contains some contradictory findings as to how macroautophagy is affected during aging, some patterns begin to emerge suggesting that one or more aspects of the autophagic machinery are changed or impaired with age. One source of variation between studies is the age of the animal one considers as aged. For example, in mouse studies, this age can vary from 16-17 months old to 22-24 months old animals. Moreover, many changes are observed between very young and aged animals but not between adult and aged animals, raising the question as to what should be considered an aging-related versus a maturation-related change. Last, conflicting results may be attributed to analyzing different brain areas, as it is conceivable that both mTOR-dependent and –independent signaling pathways acting upstream of autophagy regulation may be differentially affected by aging in different brain regions or brain cell populations.

In line with this, one unmet challenge stems from the heterogeneity of brain cells, especially in mammalian brains. Neurons in particular, comprise an extraordinary spectrum of populations with morphological and functional differences, each representing a unique “cellular context”. The genetic studies discussed earlier have clearly demonstrated that different neurons cope differently with defective macroautophagy. This may reflect the fact that macroautophagy may be endowed with a different cargo in each population, or may have adapted to distinct synaptic functions, that are neuronal-type specific. Understanding the roles and regulation of macroautophagy in different neuronal populations will greatly enhance our ability to assess the effects of age-inflicted autophagy deficits in the brain. Related to this, future work will surely provide more in-depth insight on the effects of aging on the autophagic flux *in vivo*, in specific subsets of neurons or glial cells.

A second challenge stems from the molecular diversity of the macroautophagy machinery and of its selective cargo, which has so far not been taken fully into account when assessing macroautophagy in the aged brain. For example, starting with the machinery itself, in mammals, there are at least seven Atg8 orthologs: LC3A, (with 2 splicing variants, LC3A-a and LC3A-b), LC3B, LC3C, GABARAP, GABARAPL1, and GABARAPL2. Accumulating evidence from work mainly in cancer cell lines begins to unravel the specific recruitment of these different Atg8 proteins in phagophores facilitating the sequestration of selective cargo, such as for example the role of LC3C in piecemeal mitophagy [149] versus the role of LC3A in Parkin-independent mitophagy [150]. So far, all assessment of autophagy changes in the mammalian brain with aging rely on LC3B-centric assays, with all other Atg8 proteins remaining unexplored. It remains unknown if neurons have AVs with different molecular profiles which are dedicated to different cargos or respond to different conditions and cellular states. Such information on the AV heterogeneity of brain cells would allow us to better characterize the dysregulation of autophagy in the aged brain and its implications for the homeostasis of different cargos. Similarly, most studies confer conclusions on aging-induced changes on the autophagic flux based on the accumulation of p62, a receptor for aggregates, largely ignoring the growing list of other selective receptors. Therefore, it is possible that one form of selective autophagy, such as mitophagy, is deregulated during aging, while other forms remain intact or even partially compensate. Future work will surely discriminate such potential differences to further clarify the effects of aging on selective macroautophagy in the brain. 

Another important consideration is the growing evidence for autophagy-independent functions of key components of the macroautophagy machinery, as recently reviewed [151, 152]. For example, several Atg proteins, including Atg3, Atg5 and Atg7 are involved in exosome secretion, while others, such as Beclin-1 are involved in endocytosis and other functions. Therefore, changes in steady-state levels of autophagy proteins and receptors, which are currently accepted as indicative of changed autophagic flux, may be misleading and hiding a deregulation in other cellular pathways. In neurons, autophagy-independent roles of autophagy proteins are largely unexplored to date and if they also exist they will likely change the way we interpret aging-related findings.

**Figure 3:**
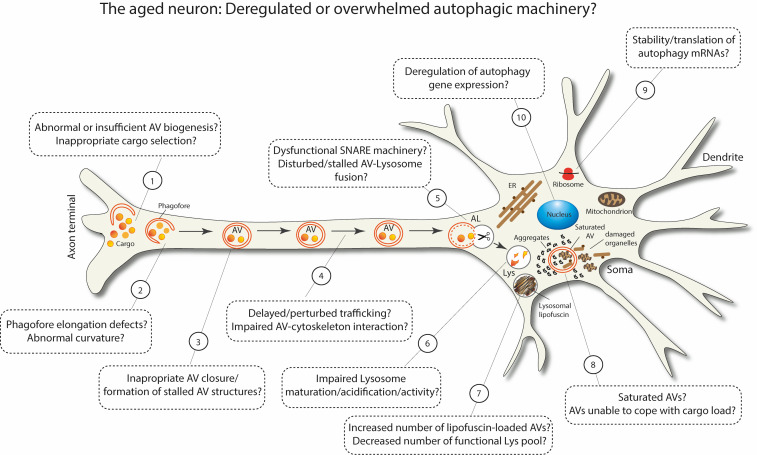
FIGURE 3: Schematic representation postulating the possible scenarios of deregulated or overwhelmed macroautophagy machinery in aged neurons.

As summarized in **Figure 3**, aging can deregulate or overwhelm different stages of the multi-step process of macroautophagy. Some of these defects have been experimentally observed in aged neurons, while others are still speculative. It is plausible that the more defects are accumulated in different steps of macroautophagy, the closer the brain comes to a threshold, beyond which macroautophagy fails and neuronal integrity deteriorates. Taken together, the recent findings in this field certainly highlight the fact that macroautophagy stands out as a key target for maintaining healthy homeostasis in the aged brain.

## Abbreviatons:

AgRP: – Agouti-related protein;
AMPA: – α-amino-3-hydroxy-5-methyl-4-isoxazolepropionic acid;
AV: – autophagic vesicle;
CMA: – chaperone-mediated autophagy;
dFP: – dual fluorescent protein;
DG: – dentate gyrus;
ER: – endoplasmic reticulum;
LTD: – long-term depression;
MB: – mushroom body;
mFP: – monomeric fluorescent protein;
MSH: – melanocyte-stimulating hormone;
mTOR: – mammalian target of rapamycin;
NMDA: – N-methyl-D-aspartate;
POMC: – proopiomelanocortin;
UPS: – ubiquitin-proteasome system;
v-ATPase: – vacuolar ATPase.
